# Supplementary material for: Electronegative LDL Promotes Inflammation and Triglyceride Accumulation in Macrophages
Source: Cells. 2020 Mar 1;9(3):583. doi: 10.3390/cells9030583 (PMC7140452; doi:10.3390/cells9030583)
Supplement: Supplementary file 1 [file cells-09-00583-s001.pdf]

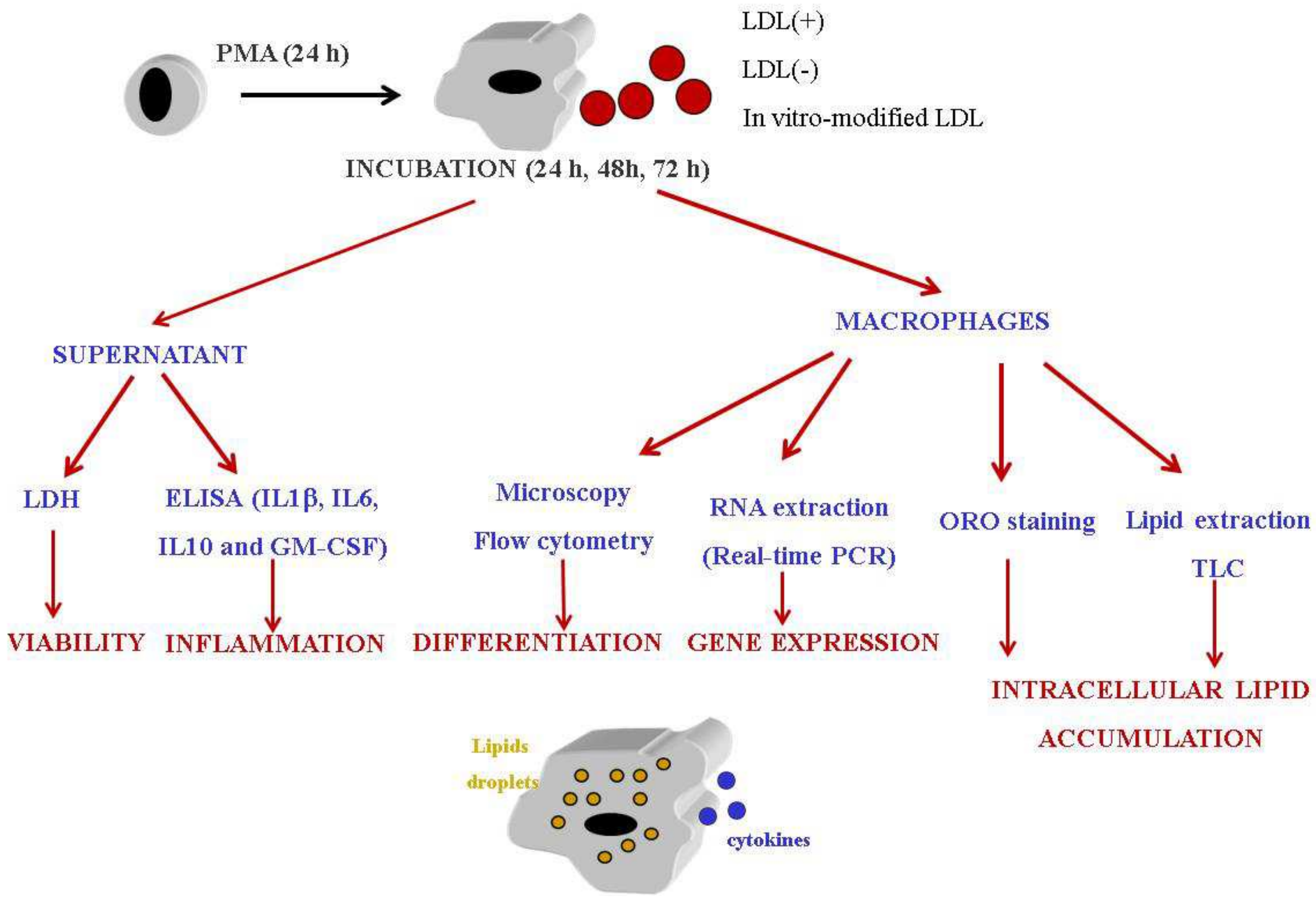

Figure S1. Experimental design. After incubation of THP1-CD14 with LDLs, supernatant was collected to mesure viability (LDH activity) and inflammation (release of inflammatory cytokines). Macrophage differentiation was evaluated by mycroscopy and flow cytometry. Intracellular lipid accumulation was evaluated by ORO staining or lipid separation (by TLC). Alternatively, cells were collected to extract RNA and quantify gene expression by real-time PCR

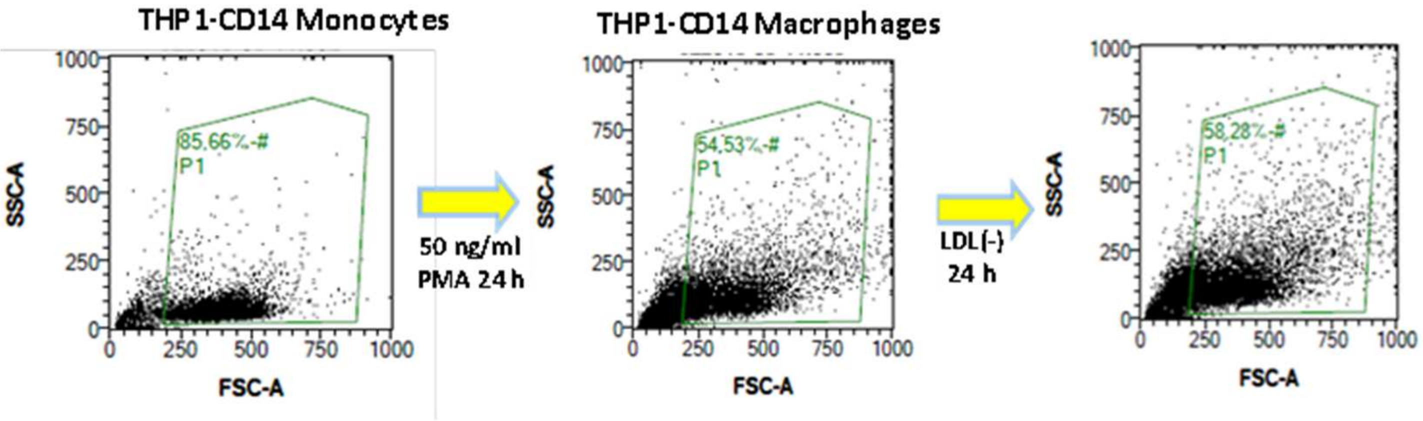

Figure S2. Representative flow cytometry analysis. Flow cytometry analysis showing size (FSC) and complexity (SSC) (SSC/FSC gating) of THP1-CD14 monocytes and macrophages. Macrophage analysis is shown in the absence and presence of LDL(-). Representative of n=4

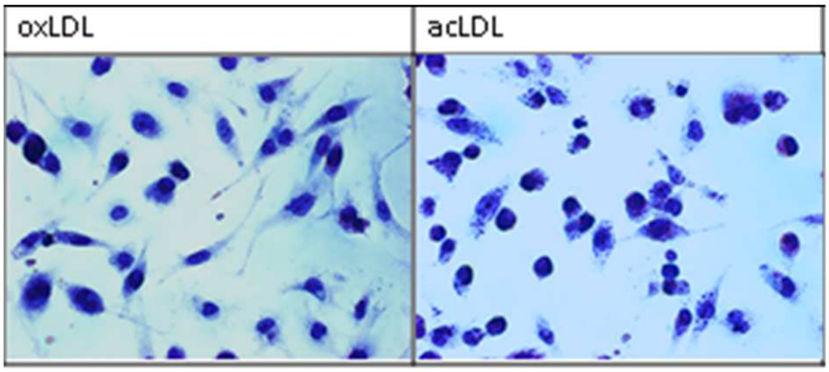

Figure S3. LD staining by oxLDL and acLDL. THP1-CD14 macrophages were seeded in 6-well plates (2 ml, 400,000 cells/ml) and incubated with oxLDL or acLDL (60 mg apoB/L) for 48 h. Afterwards, cells were fixed, stained with ORO, and observed by light-field microscopy (249.00x186.04 μm). Representative image of n=2

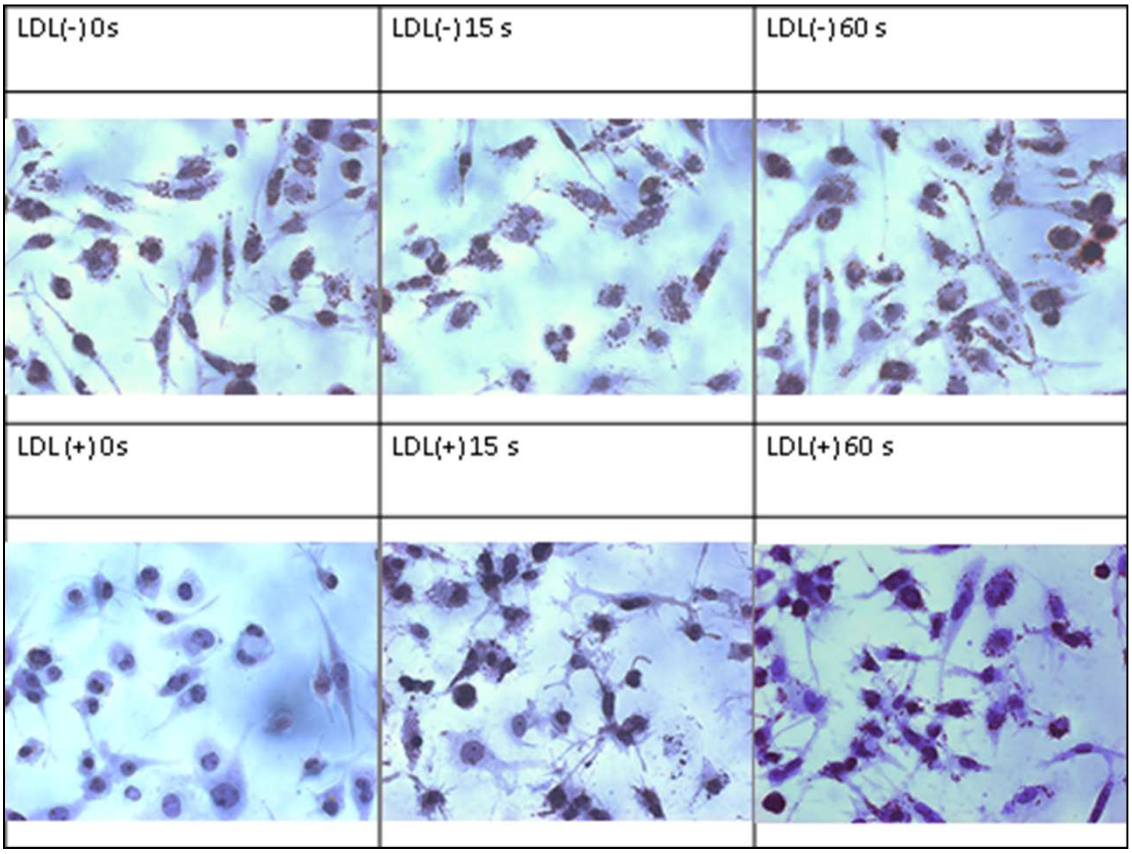

Figure S4. LD staining by aggregation of LDL(+) and LDL(-). THP1-CD14 macrophages were seeded in 6-well plates (2 ml, 400,000 cells/ml) and incubated with LDL(+) or LDL(-) (60 mg apoB/L) aggregated by vortexing (0 s, 15 s and 60 s) for 48 h. Afterwards, cells were fixed, stained with ORO, and observed by light-field microscopy (249.00x186.04 μm). Representative image of n=2

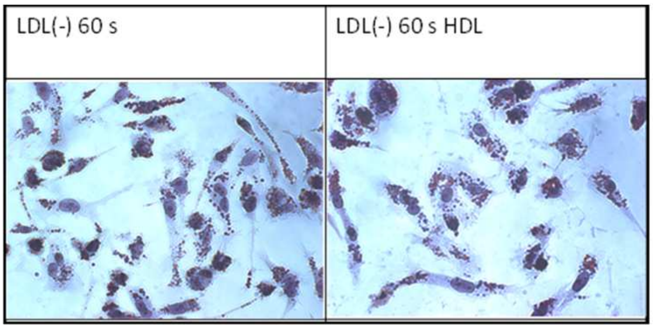

Figure S5. LD staining by LDL(-) in the presence or absence of HDL. THP1-CD14 macrophages were seeded in 6-well plates (2 ml, 400,000 cells/ml) and incubated with LDL(-) (60 mg apoB/L) in the presence or absence of HDL for 48 h. Afterwards, cells were fixed, stained with ORO, and observed by light-field microscopy (249.00x186.04 μm). Representative image of n=2

Table S1. Aggregation level of LDL(+), LDL(-), and aggLDL (absorbance at 450 nm)

	Absorbance 450 nm
LDL(+)	0.041 ± 0.004
LDL(-)	0.064 ± 0.010
aggLDL (60 s)	0.220 ± 0.123
